# Burnout in medical residents - a prospective study in Albania

**DOI:** 10.1192/j.eurpsy.2021.1934

**Published:** 2021-08-13

**Authors:** V. Alikaj, V. Skendi, E. Metaj, E. Dashi

**Affiliations:** 1 Department Of Neuroscience, University Hospital Center “Mother Theresa”, Tirane, Albania; 2 Faculty Of Medicine, Tirana Medical University Albania, Tirane, Albania

**Keywords:** resident, medical burnout, burnout consequences, burnout

## Abstract

**Introduction:**

Burnout is a syndrome characterized by the high workload in the workplace, which is very common in hospital settings. Medical trainees and early career physicians are more likely to experience burnout than their non-medical peers. Burnout has been linked with a great number of consequences, whether personal, family or work related. Physicians burnout specifically, is related to high rates of medical errors, lack of professionalism, decreased productivity but also to suicidal ideation, depression and substance abuse.

**Objectives:**

The aim of this study is to investigate the level of burnout in medical residents at University Hospital Center “Mother Theresa” Tirana, changes in burnout depending from the year of study, specialty or associated demographic factors.

**Methods:**

This is a prospective study conducted over two time periods, in 2017 and 2019 using the Maslach Burnout Inventory - short version questionnaire. The information was obtained through the direct filling in of the printed questionnaires, by the residents in their workplace.

**Results:**

We collected 137 responses from different medical specialties where 15,3% were psychiatric residents. About 70 % of residents are females and 40% of residents where in their third year of residency by the time they completed the questionnaire. 68% of residents declared more than one night shift within a week.

**Conclusions:**

Residents are given great responsibility coupled with low levels of control, placing them at risk for role problems such as role ambiguity, role conflicts or role overload. Moreover, medical residents are relatively young and at the beginning of their careers, which makes them vulnerable to burnout.

**Disclosure:**

No significant relationships.

